# Immigration, Transition into Adult Life and Social Adversity in Relation to Psychological Distress and Suicide Attempts among Young Adults

**DOI:** 10.1371/journal.pone.0046284

**Published:** 2012-10-03

**Authors:** Kyriaki Kosidou, Clara Hellner-Gumpert, Peeter Fredlund, Christina Dalman, Johan Hallqvist, Göran Isacsson, Cecilia Magnusson

**Affiliations:** 1 Division of Public Health Epidemiology, Department of Public Health Sciences, Karolinska Institute, Stockholm, Sweden; 2 Department of Clinical Neuroscience, Division of Psychiatry, Karolinska Institute, Karolinska University Hospital, Stockholm, Sweden; 3 Department of Public Health and Caring Sciences, Uppsala University, BMC, Uppsala, Sweden; Indiana University and Moi University, United States of America

## Abstract

**Background:**

The increasing incidence of mental health problems among young people is a major concern in many Western countries. The causal mechanisms underlying these trends are not well established, but factors influenced by current societal changes ought to be implicated. Such factors include immigration and social adversity as well as the timing of taking on adult social roles (e.g. gainful employment, parenthood and own housing tenure). We therefore examined relationships between these factors and the risks of psychological distress as well as suicide attempts in young adults, with a focus on gender differences.

**Methods:**

We conducted a population-based study including 10,081 individuals aged 18–29, recruited in 2002 and 2006 in Stockholm, Sweden. Data were collected by record linkage and questionnaires.

**Results:**

Non-European immigrants had an increased risk of distress, and female non-European immigrants had a markedly higher risk of suicide attempts. Both early parenthood (≤24 years) and not being a parent, being a student and the lack of own housing tenure were associated with distress, but only in women. In both sexes, financial strain was associated with the increased risk of distress and suicide attempts, while unemployment was only associated with distress.

**Conclusions:**

Immigration from outside Europe and social adversity are associated with mental health problems in young adults, especially females. Postponed transition into adulthood is associated with poor mental health in young women. These factors are influenced by current societal changes, and may have contributed to the increasing incidence of mental health problems among young people in Western countries.

## Introduction

Young adulthood is the peak age for the onset of most mental disorders and is a period of crucial importance for the establishment of emotional well-being in adult life [1], [2], [3]. Mental health problems, including psychological distress and depressive symptoms [4], [5], [6], [7], [8], [9] as well as suicide attempts [4], [10], [11], [12] are reported to be increasing among young people, especially females, in many Western countries. Little is known about the socio-demographic distribution and causes of this apparent epidemic, although factors influenced by societal changes are likely to be implicated. Such changes include increasing migration rates [13] and social disadvantages such as youth unemployment and poverty [14]. Immigrant populations have more than tripled in most western countries since the 1960s [15] and in Sweden in 2011, about 15% of the population was born abroad. In the European Union over the past decade, youth unemployment has been about twice as high as that of the total population (http://epp.eurostat.ec.europa.eu/statistics_explained/index.php/Unemployment_statistics.). Furthermore, the transition into adult life has also changed considerably during recent decades, with the attainment of employment, family and housing being delayed to an older age, probably reflecting increasing labour market demands for an educated workforce, shortage of rental housing and women’s emancipation as well as other cultural changes [16], [17], [18], [19], [20]. Such societal and cultural changes may affect the mental health of young people, but their contributions to the growing occurrence of ill health are not well known. We therefore studied the relationships between immigrant status, transition into adult life (as reflected by employment status, age at becoming a parent and housing tenure), social adversity (including unemployment and financial strain) and psychological distress as well as suicide attempts (two dimensions of mental ill health that are reportedly increasing), in a large population-based sample of young adults. Since these factors may influence mental health differently between young men and young women, the latter group having a higher prevalence of both psychological distress and suicide attempts, our study focused on possible gender differences.

## Methods

### Study Population

The study population consisted of those men and women aged 18–29 who participated in the 2002 (N = 4,911) and 2006 (N = 5,170) survey waves of the Stockholm Public Health Cohort [21]. The surveys used area- and, in 2002, also sex-stratified, random samples of the population of Stockholm County aged 18–84 years. The sample was drawn from the Total Population Register, a register held by Statistics Sweden that records basic demographic and socioeconomic information for the total population in Sweden. An equal number of individuals were sampled per municipality, resulting in a total sample size of 49,909 and 56,634 for 2002 and 2006 respectively. The sample size was tailored to enable adequately powered estimations of age-, sex- and municipality-specific prevalence of core health determinants. Data were collected using postal or web-based questionnaires that elicited information on socio-demographic factors, psychological distress, suicide attempts as well as other health and life-style characteristics. Self-reported exposure and outcome information was complemented by information from longitudinal health and socio-demographic data registers, regarding study participants as well as their first-degree relatives. The key to our ability to link these registers was the unique personal identification number assigned to each resident of Sweden. Response rates among individuals aged 18–29 were 54% in 2002 and 51% in 2006, respectively. Non-responders were more likely to be men, born outside Sweden, single or separated, unemployed and with lower incomes [22]. Written informed consent was obtained from all study participants and the Stockholm regional ethical review board granted approval for the study.

### Measures

Information about country of birth was attained by linkage with the Longitudinal Integration Database for Health Insurance and Labour Market Studies (LISA), held by Statistics Sweden (http://www.scb.se). We categorized immigrant status as native Swede, European and Non-European first generation immigrant (those born outside Sweden in Europe and elsewhere, respectively), and European and Non-European second generation immigrant (Swedish-born to parents with origins outside Sweden in Europe or elsewhere, respectively). In both surveys, the participants reported their current employment status, which was categorized as: student, employed, unemployed and other (housewives, sick leave, disability pension or parental leave). Financial strain was assessed via the questions: (i) “In the past 12 months have you spent your entire paycheque/pension or run out of money and been forced to borrow from relatives and friends in order to buy groceries or pay the rent?” and (ii) “In the past 12 months have you spent your entire paycheque or run out of money and been forced to turn to social services in order to buy groceries or pay the rent?” For both questions there were three response alternatives: “No”, “Yes, once”, “Yes, many times”. These were combined to create a three-category variable: no financial strain; sought financial help from others; sought social benefits. Data on study participants’ age at becoming a parent were collected from the Swedish Multigenerational Register [23] and grouped as: 14–19 years of age, 20–24 years of age, 25–29 years of age, and nonparent. Housing tenure was measured with two questions: (i) “What kind of housing do you live in?” and (ii) “With whom do you share your housing?” Data were grouped in three categories: owning/renting (including owners and first hand renters); living with family of origin (including parents and siblings); lacking own tenure (including second hand tenures, lodgers and those in student accommodation).

Psychological distress was assessed via the 12-item version of the General Health Questionnaire (GHQ-12) [24]. The GHQ-12 has been validated for use in the Swedish population [25] and a cut-off score of ≥3 (using the recommended standard 0-0-1-1 scoring) is generally used in public health surveys and research reports to denote significant psychological distress, consistent with the presence of a common mental disorder [25], [26], [27]. Lifetime suicide attempts were assessed by a question based on the work of Meehan [28]: “Have you ever made an attempt to take your life?” There were four answer alternatives: “No, never”, “Yes, in the last week”, “Yes, in the last year”, “Yes, more than a year ago”. Responders choosing the three positive alternatives were considered as having had lifetime suicide attempts. “Suicide attempt” is the usual expression among laymen. Since we did not address the question of lethal intent, the expression should be interpreted as deliberate self-harm.

### Potential Confounders

Information on parental socio-economic status (SES) was obtained through linkage to Statistics Sweden and the Swedish Population and Housing Census of 1990. Register-based data were supplemented by self-reported information on parental SES, available from the 2002 survey. Parental SES was defined as the highest SES of the mother or father, classified according to the *Swedish socio-economic classification* developed by Statistics Sweden [29] as: unskilled workers, skilled workers, lower non-manual employees, intermediate non-manual employees, higher non-manual employees and self-employed. Data on parental education was obtained by linkage with LISA and grouped in three categories: compulsory (0–9 years in duration), upper secondary (10–12 years in duration) and higher (>12 years in duration), according to the highest educational achievement of the mother or father. School performance was defined as grade point averages in the final year of compulsory education (year 9, when participants were approximately 16 years old), retrieved from the National School Register at the Swedish National Agency for Education (available at http://www.skolverket.se), and categorized into quintiles according to year of graduation. Information on hospital admissions for mental disorders (any diagnosis in Chapter V of ICD-8 and 9 or Chapter F of ICD-10) in study participants and their parents was obtained from the Swedish National Patient Register (available at http://www.socialstyrelsen.se). A positive psychiatric history was defined as any admission prior to completing the surveys in participants, and at any time in parents.

### Statistical Analysis

Analyses were conducted using SAS version 9.1 (SAS Institute Inc., Cary, N.C., USA). In order to increase statistical power for the analyses, the 2002 and 2006 samples were pooled. There were 76 individuals that had participated in both surveys, and these were excluded from the analyses. The final analytical sample consisted of 10,081 individuals. Statistical methods for reweighting for non-response can be applied when non-respondents can be appropriately characterized, as is the case in Sweden where extensive total population registers are available [30]. Calibration weights, designed to re-calculate the population structure with compensation for systematic non-response, have been created for each survey by Statistics Sweden and were used in this study. The weights are constructed on the basis of available auxiliary variables from national registries and their co-variation with survey data. The auxiliary variables include sex, age, country of birth, civil status, income, educational level, sickness allowance and area of residence. We carried out logistic regression analyses to estimate crude and adjusted odds ratios (OR) and their 95% confidence intervals (CI) of psychological distress and suicide attempts in relation to immigrant status, employment status, financial strain, age at becoming a parent and housing tenure. All analyses were adjusted for age, and then further in multivariate models such that: (i) immigrant status was adjusted for parental SES, parental education and parental history of inpatient psychiatric care; and (ii) indicators of social adversity and transition into adult life were adjusted for parental SES, parental education, immigrant status, school performance, parental and individual history of inpatient psychiatric care as well as for each other. Analyses were conducted separately among men and women. In order to evaluate whether factors were differentially associated with psychological distress and suicide attempts in men and women, we calculated the synergy index (SI) [31]. The SI is a measure of departure from additivity, which reflects whether a joint effect is greater than the sum of the independent effects (of gender and the respective exposures). The SI is defined as [OR_11_−OR_00_]/[(OR_01_−OR_00_)+(OR_10_−OR_00_)], where the first and second index digits indicate the absence or presence of the two respective risk factors under study. The index denotes synergy if its value exceeds 1.0 and antagonism if it is less than 1.0.

## Results

Characteristics of the study participants are presented in Table 1. Psychological distress and lifetime suicide attempts were respectively about 50% and twice as common in women as in men.

**Table 1:** Characteristics of the study participants (N = 10,081).

Characteristics	Men (N = 4,311) %	Women (N = 5,770) %
**Age group**		
18–20	16.8	15.3
20–24	35.6	35.8
25–29	47.6	48.9
**Immigrant status**		
Non-immigrant	60.3	59.2
1^st^ generation immigrant born in Europe	4.5	5.6
1^st^ generation immigrant born outside Europe	9.4	9.5
2^nd^ generation immigrant from Europe	11.6	12.1
2^nd^ generation immigrant outside Europe	14.2	13.7
**Parental socio-economic status**		
Unskilled workers	9.2	9.2
Skilled workers	10.5	10.5
Lower non-manual employees	12.9	12.1
Intermediate non-manual employees	24.6	23.4
Higher non-manual employees	28.0	26.7
Self-employed	14.9	18.1
**Parental education**		
Compulsory	9.6	10.9
Upper secondary	44.4	45.8
Higher	46.0	43.3
**Employment status**		
Student	32.7	33.3
Employed	55.8	51.4
Unemployed	6.7	6.5
Other	4.7	8.8
**Financial strain**		
No financial strain	69.1	62.3
Sought financial help from others	27.8	33.4
Sought social benefits	3.1	4.3
**Age at becoming a parent**		
14–19	0.2	1.4
20–24	3.8	8.1
25–29	5.7	7.6
Non-parent	90.3	82.9
**Housing tenure**		
Owning/renting	51.5	60.4
Living with family of origin	37.2	27.0
Lacking own housing tenure	11.4	12.7
**School performance**		
Lowest quintile	26.0	17.0
2^nd^ quintile	23.9	19.1
3^nd^ quintile	19.8	20.5
4^th^ quintile	17.0	21.1
Highest quintile	13.3	22.4
**Prevalence of mental health problems**		
Lifetime suicide attempts (%)	4.2	8.6
Psychological distress (GHQ-12≥3) (%)	24.0	36.4

Psychological distress was somewhat more common in non-European second generation immigrants of both sexes, as compared to native Swedes (Table 2). In women, first generation non-European immigrants also had a higher risk of distress. For suicide attempts, the gender difference in the relationship between immigration status and suicide attempts was even more marked, and we noted a strong synergistic effect of being both female and of non-European origins (SI 4.58, 95% CI 1.97–10.67). Non-European first generation immigrant women had a more than 3-fold elevated risk of suicide attempts (OR 3.52, 95% CI 2.61–4.74) as compared to native Swedes, while the excess risk among second generation immigrant women was less pronounced (OR 1.60, 95% CI 1.23–2.10). In contrast, there was no association between immigrant status and suicide attempts in men.

**Table 2:** Odds ratios (OR) and 95% confidence intervals (CI) of psychological distress and suicide attempts in relation to immigrant status among young adults in the Stockholm Public Health Cohort.

Psychological distress						
		Men			Women	
	Cases/non- cases	Base Model¶	Full Model†	Cases/non-cases	Base Model¶	Full Model†
	N	OR (95%CI)	OR (95%CI)	N	OR (95%CI)	OR (95%CI)
**Immigrant status**						
Native Swede	551/1883	1	1	1136/2101	1	1
European 1^st^ generation immigrant	24/75	1.08 (0.76–1.52)	1.04 (0.73–1.49)	56/111	0.98 (0.74–1.31)	1.03 (0.76–1.38)
Non-European 1^st^ generation immigrant	44/129	1.07 (0.82–1.39)	1.11 (0.84–1.46)	126/170	1.37 (1.11–1.69)*	1.41(1.13–1.75)*
European 2^nd^ generation immigrant	98/366	0.96 (0.78–1.17)	1.00 (0.81–1.22)	231/412	1.01 (0.85–1.20)	1.00 (0.84–1.19)
Non-European 2^nd^ generation immigrant	148/393	1.21 (1.02–1.45)*	1.26 (1.05–1.51)*	288/412	1.30 (1.10–1.52)*	1.28 (1.08–1.51)*
**Suicide attempts**						
		**Men**			**Women**	
	**Cases/non- cases**	**Base Model** ¶	**Full Model** †	**Cases/non-cases**	**Base Model** ¶	**Full Model** †
	**N**	**OR (95%CI)**	**OR (95%CI)**	**N**	**OR (95%CI)**	**OR (95%CI)**
**Immigrant status**						
Native Swede	90/2304	1	1	227/2994	1	1
European 1^st^ generation immigrant	May-93	1.17 (0.57–2.42)	1.08 (0.51–2.32)	17/150	1.43 (0.88–2.31)	1.61 (0.97–2.65)
Non-European 1^st^ generation immigrant	8/160	1.01 (0.56–1.84)	0.98 (0.53–1.82)	48/240	3.35 (2.52–4.45)*	3.52 (2.61–4.74)*
European 2^nd^ generation immigrant	18/441	1.15 (0.76–1.74)	1.06 (0.70–1.61)	53/585	1.39 (1.04–1.85)*	1.30 (0.97–1.74)
Non-European 2^nd^ generation immigrant	20/508	0.88 (0.58–1.33)	0.72 (0.47–1.10)	71/621	1.78 (1.37–2.30)*	1.60 (1.23–2.10)*

¶ Adjusted for age.

† Adjusted for age, parental SES, parental education and parental history of in-patient psychiatric care.

* Significant at the.05 level, 2-sided test.

Unemployed men and women, and those reporting financial strain, had two to threefold increases in risk of distress (Table 3). Women who were students or off the labour market for other reasons also had a higher risk of distress compared to those employed, but these associations were less striking (Table 3). There was also a clear gender difference, with no evidence of any relationships in men, and statistically significant SIs for female gender and being a student or being off the labour market for other reasons (SIs 1.62, 95% CI 1.15–2.27 and 1.33, 95% CI 0.73–2.41, respectively). Women who had a first childbirth as teenagers had an OR of 3.68 (95% CI 1.88–7.17) for distress, and both women who became mothers at the age of 20–24 as well as those not having children had less marked but still statistically significantly elevated risks of distress compared to women who became mothers at the age of 25–29 (Table 3). Furthermore, age- and gender-stratified analysis showed that among women of 25–29 years old, nulliparous women had an OR of 1.71 for distress (95% CI 1.25–2.36) as compared to those whose first childbirth was at the age of 25–29. Parenthood did not, however, seem to be related to men’s distress, although SIs were not statistically significant (SIs 2.42, 95% CI 0.31–18.74 and 3.83, 95% CI 0.41–35.94 for female gender and parenthood before the age of 25 and nonparents, respectively). The lack of one’s own housing tenure slightly increased the risk of distress in women but not in men (SI 1.52, 95% CI 1.01–2.28). Finally, financial strain was associated with an increased risk of suicide attempts in both sexes, but the other aspects of social adversity or the taking on of adult social roles were unrelated to such attempts (Table 4).

**Table 3:** Odds ratios (OR) and 95% confidence intervals (CI) of psychological distress in relation to social adversity and transition into adult life among young adults in the Stockholm Public Health Cohort.

		Men			Women	
	Cases/non-cases	Base Model¶	Full Model†	Cases/non-cases	Base Model¶	Full Model†
	N	OR (95%CI)	OR (95%CI)	N	OR (95%CI)	OR (95%CI)
**Employment status**						
Student	263/860	1.22 (1.04–1.43)*	1.03 (0.87 1.22)	592/902	1.41 (1.23–1.61)*	1.33 (1.16–1.54)*
Employed	416/1547	1	1	810/1662	1	1
Unemployed	86/114	3.18 (2.49–4.07)*	2.58 (1.99–3.34)*	149/128	2.57 (2.01–3.28)*	2.30 (1.78–2.96)*
Other	39/123	1.34 (0.98–1.83)	1.20 (0.87–1.65)	142/233	1.33 (1.07–1.66)	1.42 (1.11–1.81)
**Financial strain**						
None	478/1964	1	1	913/2001	1	1
Sought help from others	295/649	1.97 (1.71–2.27)*	1.93 (1.67–2.24)*	695/864	1.70 (1.51–1.93)*	1.55 (1.37–1.76)*
Sought social benefits	31/31	3.75 (2.44–5.78)*	3.11 (1.96–4.92)*	85/60	2.82 (2.06–3.87)*	1.92 (1.37–2.71)*
**Age at becoming a parent**						
14**–**19	1/5	1.61 (0.34–7.62)	1.31 (0.27–6.37)	26/18	4.91 (2.58–9.34)*	3.68 (1.88–7.17)*
20**–**24	27/79	1.36 (0.82–2.26)	1.26 (0.75–2.12)	114/196	1.85 (1.30–2.62)*	1.54 (1.07–2.21)*
25**–**29	40/168	1	1	90/252	1	1
Non-parent	736/2392	1.30 (0.94–1.81)	1.13 (0.80–1.58)	1463/2459	1.68 (1.27–2.21)*	1.73 (1.29–2.34)*
**Housing tenure**						
Owning/Renting	408/1399	1	1	993/1809	1	1
Living with family of origin	294/952	1.28 (1.07–1.54)*	1.18 (0.97–1.44)	439/781	0.97 (0.82–1.15)	0.94 (0.78–1.12)
Lacking own tenure	102/293	1.26 (1.02–1.55)*	1.11 (0.89–1.38)	261/335	1.36 (1.14–1.61)*	1.24 (1.04–1.49)*

¶ Adjusted for age.

† Adjusted for age, parental SES, parental education, immigrant status, school performance, employment status, financial strain, age at becoming a parent, housing tenure, parental and individual history of in-patient psychiatric care when applicable.

* Significant at the.05 level, 2-sided test.

**Table 4:** Odds ratios (OR) and 95% confidence intervals (CI) of suicide attempts in relation to social adversity and transition into adult life among young adults in the Stockholm Public Health Cohort.

		Men			Women	
	Cases/non-cases	Base Model¶	Full Model†	Cases/non-cases	Base Model¶	Full Model†
	N	OR (95%CI)	OR (95%CI)	N	OR (95%CI)	OR (95%CI)
**Employment status**						
Student	37/1069	0.72 (0.50–1.03)	0.84 (0.57–1.24)	103/1379	0.89 (0.69–1.13)	1.12 (0.86–1.47)
Employed	73/1849	1	1	181/2274	1	1
Unemployed	12/187	1.47 (0.88–2.44)	0.98 (0.56–1.70)	40/236	2.22 (1.58–3.13)*	1.19 (0.79–1.77)
Other	7/152	1.21 (0.65–2.23)	1.17 (0.62–2.21)	44/329	1.74 (1.24–2.43)*	1.07 (0.72–1.59)
**Financial strain**						
None	62/2335	1	1	155/2737	1	1
Sought help from others	58/871	2.40 (1.77–3.24)*	2.04 (1.49–2.81)*	166/1387	2.18 (1.75–2.71)*	1.80 (1.42–2.27)*
Sought social benefits	9/51	4.96 (2.48–9.91)*	1.70 (0.78–3.82)	47/94	9.18 (6.40–13.17)*	3.57 (2.29–5.55)*
**Age at becoming a parent**						
14**–**19	0/6	<<<<	<<<<	11/33	3.29 (1.54–7.04)*	0.83 (0.63–1.92)
20**–**24	11/92	2.92 (1.31–6.51)*	2.10 (0.90–4.89)	44/263	1.73 (1.03–2.92)*	0.91 (0.52–1.62)
25**–**29	9/198	1	1	26/316	1	1
Non-parent	109/2961	0.70 (0.36–1.33)	0.93 (0.47–1.85)	287/3606	0.73 (0.47–1.14)	0.89 (0.54–1.47)
**Housing tenure**						
Owning/Renting	67/1705	1	1	233/2552	1	1
Living with family of origin	47/1173	1.01 (0.67–1.52)	1.05 (0.68.1.62)	92/1115	0.82 (0.61–1.10)	0.78 (0.57–1.09)
Lacking own tenure	15/379	1.03 (0.64–1.66)	1.16 (0.70–1.91)	43/551	0.83 (0.61–1.14)	0.80 (0.56–1.15)

¶ Adjusted for age.

† Adjusted for age, parental SES, parental education, immigrant status, school performance, employment status, financial strain, age at becoming a parent, housing tenure, parental and individual history of in-patient psychiatric care when applicable.

* Significant at the.05 level, 2-sided test.

## Discussion

Findings from this large population-based study show that immigration from outside Europe is associated with poor mental health in young people, and especially in young women. The striking risk increase for suicide attempts in non-European immigrant women is of particular concern. Furthermore, postponement of adulthood, as reflected by later attainment of employment and housing tenure as well as becoming a parent at a later age, appear to be related to poor mental health, but only in young women. Lastly, social adversity, particularly financial strain, is strongly related to mental ill health in young people regardless of gender.

Our finding that suicide attempts were more common among women, but not men, who were first or second generation non-European immigrants, is in line with one recent study from the Netherlands [32]. That study found an elevated risk of suicide attempts among young women, but not men, of Turkish ancestry. A Swedish record-linkage study demonstrated a positive relationship between immigration and hospital admissions for self-inflicted injury, but did not find significant gender differences [33]. Our finding that non-European immigrants had a higher risk of distress, regardless of gender, corroborates that of a Belgian study [34]. As this study examined adults regardless of age, our study adds to prior research by showing a high prevalence of psychological distress among younger non-European immigrants. Young people facing unfavourable socio-economic circumstances like financial strain and unemployment had a high risk of distress in our study, which is in line with prior reports [1], [35], [36]. The association between economic disadvantage and suicidal behaviour in young people has been well documented [37] and is confirmed in our study among both men and women. In contrast, the relationships between employment status, age of parenthood and housing tenure and mental health differed between men and women. Our findings support those of an Australian study that demonstrated improvement in mental health among young women transitioning from studying to paid employment while the opposite was true for the inverse transition and for those that were unemployed [38]. Women giving birth at an early age as well as nulliparous women reported higher levels of psychological distress than those with a first birth at the age of 25–29. This finding is in line with some earlier reports that the effect of age at first birth on mental health for women is curvilinear, with first births at both young and older ages (i.e. after age 30) being positively associated with psychological distress [39], [40].

This study’s strengths include its large population-based sample and the combined use of self-reported and register-based data. We used validated instruments to identify cases of psychological distress [25] and suicide attempters [28]. We were able to adjust for confounding effects of severe parental and individual psychopathology assessed from valid psychiatric care registers [41], [42]. There are, however, also limitations. Causal inference regarding our findings for employment status, financial strain and housing tenure is problematic, since it is based on cross-sectional analyses. We studied young individuals, who are least likely to participate in surveys [43]. Although we used weights to adjust for non-participation, selection bias may have influenced our results to some extent. Furthermore, analyses of suicide attempts were sometimes based on small numbers. Lastly, we did not have information on all possible determinants of mental ill health in young adulthood, e.g. childhood/teenage psychopathology or abuse, and thus confounding cannot be ruled out.

The strong relationship between non-European origin and suicide attempts in young women could not be explained by parental or individual socio-economic conditions in these data. Many of the non-European immigrants are refugees and it cannot be determined to what extent traumatic experiences before coming to Sweden and circumstances in Sweden, respectively, may contribute. It may be that young non-European immigrant women face adverse living conditions and possibly cultural and family conflicts not shared by their male peers, which may be of relevance for prevention. Previous research suggests that such intergenerational conflicts as well as social marginalization and discrimination [44], [45], [46] may mediate the relationship between immigration and mental ill health. Cultural differences may predict such adverse phenomena, which might explain why only non-European immigrant women had a higher risk of distress and attempted suicides in our study.

Financial strain and unemployment were positively related to psychological distress in both sexes in our study. Thus living conditions, job opportunities and the socio-economic environment in which young people establish themselves early in life are important for mental health. Furthermore, financial strain was also associated with the risk of suicide attempts, indicating that poverty is an important determinant of suicidal behaviour in young people. Our results may also reflect reverse causality, i.e. that mental ill-health hampers gainful employment and hence prosperity, since they are based on cross-sectional analyses. It should, however, be noted that the relationships remained after adjustment for severe mental illness, which is why financial strain may have a causal role in the development of mental ill health in young people. Furthermore, although mental health problems could lead to financial strain via unemployment, unemployment per se was not independently associated with suicide attempts in our study. This finding contradicts some previous reports that found a correlation between unemployment and suicidal behaviour in young people [47], [48], [49]. It might be that poverty and other confounding factors explain much of the connection between unemployment and suicidal behaviour found in previous studies [50], or that the extensive Swedish social security system characterized by large expenditures on active labour-market programs and unemployment benefits partly alleviates the impact of unemployment on the mental health of young people in this country.

Entering the work force, attaining residential independence and becoming a parent are core role transitions signalling the entry into adulthood. In our study, postponed transition to such adult roles was associated with poor mental health, but only among women. Correlates like physical health, unfulfilled expectations about family forming and deviations from culturally expected birth timing may all mediate the association between not giving birth before the age of 30 and distress. It may also be that taking on mature social roles and responsibilities such as parenthood and employment improves emotional well-being especially in women. This mechanism could possibly also explain why women lacking their own housing tenure had higher levels of distress, after adjustment for possible socio-economic confounders. Lacking one’s own housing tenure may involve less control and responsibility in the young women’s lives and thus threaten emotional well-being. Furthermore, the attainment of paid employment and family as well as residential independence may also reflect young peoplés chances and ability to make independent reproductive, occupational and lifestyle choices. The gender differences found in our study indicate that the transition into adult life and making such choices may be especially problematic for young women who encounter difficulties and ambiguities that are not paralleled among young men.

Early motherhood was also related to poor mental health among women. Early childbearing perhaps relates to mental ill health both because it may reflect a disordered transition from adolescence into adulthood, and because it may itself disrupt that transition [51]. Early motherhood shapes the life course in ways that might diminish a woman’s capacity to develop personal resources and social capital, and hence erode emotional and physical well-being [52].

Finally, our study indicates that psychological distress and suicide attempts have partly different determinants. Psychological distress relates to a broad range of life circumstances and concurrent adversities while more serious psychiatric morbidity, as reflected by suicide attempts, relates only to some of them. However, psychological distress is itself a risk factor for suicide attempts among young people [53] and even milder forms of distress are linked to long-term disability [54]. Thus, factors influencing a young person’s risk for distress may have long-term importance even for more serious mental health problems.

In conclusion, youth unemployment and poverty are related to mental ill health in young adults and immigration from outside Europe is related to mental ill health, especially among young women. Postponement of the adoption of adult roles is also related to poor mental health in young women. All these factors reflect the way and the context in which young people pave their path early in life and are influenced by societal changes. Recent societal changes influencing these phenomena may have contributed to the increasing rates of mental health problems in young adults, especially young females, in Western countries.
